# Robotic-integrated intraoperative ultrasound for excision of accessory cavitated uterine malformation (ACUM): an innovative surgical technique

**DOI:** 10.1007/s00404-026-08395-2

**Published:** 2026-03-19

**Authors:** Smitha Priyadarshini Thippeswamy, Radwa Hablase, Joydeep Chatterjee, Raef Faris, Priya Narayanan, Jayanta Chatterjee

**Affiliations:** 1 Academic Department of Gynaecology Oncology, Royal Surrey Hospital NHS Foundation Trust, Egerton Road, Guildford, Surrey GU2 7XX UK; 2 Imperial College London, London, UK; 3 Lister Hospital, Chelsea, London, UK; 4 Department of Radiology, University College Hospital, London, UK

**Keywords:** Accessory cavitated uterine mass, ACUM, Robotic surgery, Intraoperative ultrasound, Müllerian anomaly, Dysmenorrhea, Accessory cavitated uterine malformation

## Abstract

**Background:**

Accessory cavitated uterine mass (ACUM) is a rare Mullerian anomaly that predominantly affects young women, presenting with refractory dysmenorrhea. Despite 3D ultrasound and MRI being the gold standard for diagnosis, diagnostic delays and accuracy limitations continue to hinder timely management. The deep intra-myometrial location of this anomaly poses significant surgical challenges in achieving complete excision while preserving healthy myometrium.

**Case Presentation:**

A 22-year-old nulliparous woman presented with incapacitating cyclical pelvic pain and was diagnosed with ACUM. She underwent surgical management using a novel robotic-assisted technique.

**Technique:**

The procedure utilised robotic-integrated intraoperative ultrasound via a drop-in probe, enabling real-time identification of lesion margins, vascular mapping, and confirmation of complete lesion excision. This approach minimised loss of healthy myometrium and preserved endometrial cavity integrity.

**Outcome:**

The patient reported complete symptom resolution postoperatively. Integration of real-time intraoperative ultrasound within the robotic platform enhanced surgical precision, reduced surgical trauma, and has the potential to improve reproductive outcomes.

**Conclusion:**

Robotic-assisted excision of ACUM with intraoperative ultrasound guidance represents an innovative and precise surgical technique. This approach addresses the key challenges of lesion localisation and myometrial preservation, offering a promising strategy for managing this rare condition in women of reproductive age.

## What does this study adds to the clinical work?

**Table:** 

We describe a novel robotic-integrated intraoperative ultrasound surgical technique for the management of Accessory Cavitated Uterine Mass (ACUM), addressing the challenge of precise and complete excision. This approach establishes a reproducible standard for minimally invasive management, offering precise margin definition while minimizing surgical trauma to healthy myometrium and optimizing reproductive outcomes in women with this rare Mullerian anomaly.	

## Introduction

Accessory Cavitated Uterine Malformation (ACUM) is a rare congenital Mullerian anomaly characterized by a noncommunicating accessory cavity within an otherwise normal uterus [1, 2]. ACUM is described as a solitary accessory cavitated lesion lined by functional endometrial tissue encased by smooth muscle within the myometrium, occurring in young women who otherwise have normal uterine anatomy [2, 3]. Current etiopathogenetic theories suggest two main mechanisms—an isolated Mullerian duct malformation with tissue duplication at the level of the round ligament, and gubernaculum dysfunction during embryonic development leading to persistence of ductal Mullerian tissue. Considering the characteristic anatomical location and its association with the normal uterine development, the latter theory is increasingly accepted [4]. With advances in minimally invasive surgical techniques, particularly robotic-assisted laparoscopy, management approaches for ACUM have evolved significantly over the past five years.

## Clinical details

A 22-year-old nulliparous woman presented with a history of chronic incapacitating severe cyclic pelvic and lower abdominal pain radiating down her left leg, which began at the age of 16. Initial evaluation included extensive imaging with pelvic ultrasonography, magnetic resonance imaging, and computed tomography, which suggested congenital uterine malformation consistent with a Müllerian duct anomaly. She subsequently underwent a diagnostic hysteroscopy and laparoscopy and was informed that she had a bicornuate or septate Müllerian anomaly, corresponding to a U2/U3 classification of uterine anomaly. Initially, medical management with oral progesterone and gonadotropin-releasing hormone analogues provided only temporary relief. Due to persistent severe dysmenorrhea, cyclical bleeding, and incapacitating pelvic pain that significantly affected her normal life during menstruation, she was referred to our care by her general practitioner for further evaluation and definitive management.

Written informed consent was obtained from the patient for this publication.

## Diagnosis

**Fig. 1:**
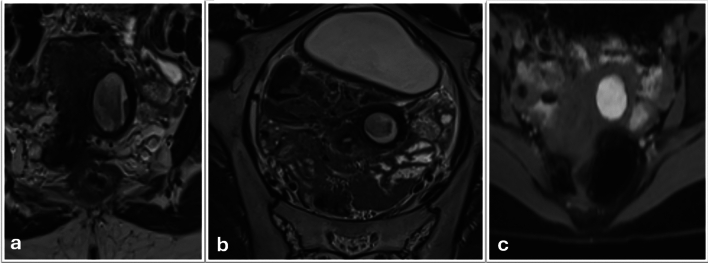
Radiological features of ACUM. **a**, **b** Coronal T2-weighted and Axial T2-weighted MRI showing left-sided myometrial mass with low T2-weighted signal rim and central intermediate to high signal. There is no communication with the endometrial cavity. **c** Axial T1-fat saturated image showing that the mass is of high T1-weighted signal intensity in keeping with hemorrhagic content

Magnetic resonance imaging (Fig. 1) revealed a solitary, well-defined cavitated lesion embedded within the lateral myometrium, inferior to the round ligament insertion. T2-weighted sequences demonstrated a rounded cystic lesion with mixed signal intensity, indicative of intracavitary hemorrhage of varying chronicity. The mass comprised a distinct myometrial rim encasing an inner lining isointense appearance to the functional endometrium. Central contents appeared hyperintense on T1-weighted images and heterogeneous on T2-weighted images, consistent with chronic hemorrhagic debris. Crucially, the uterine cavity and cornua preserved normal morphology, effectively excluding a noncommunicating rudimentary horn. Adjacent myometrium and the contralateral uterus appeared unremarkable. These radiological features supported a diagnosis of accessory cavitated uterine mass (ACUM), distinct from adenomyosis, adenomyoma, or degenerating leiomyoma.

## Surgical technique

### Robotic excision of accessory cavitated uterine mass

Following general anesthesia, the patient was positioned in a 45-degree Trendelenburg position. A standard multiport robotic Da Vinci Xi (intuitive surgical) setup was used, with an 8-mm umbilical camera port and two 8-mm robotic accessory ports placed laterally at the midumbilical level. A thorough inspection of the pelvic cavity was performed.

**Fig. 2:**
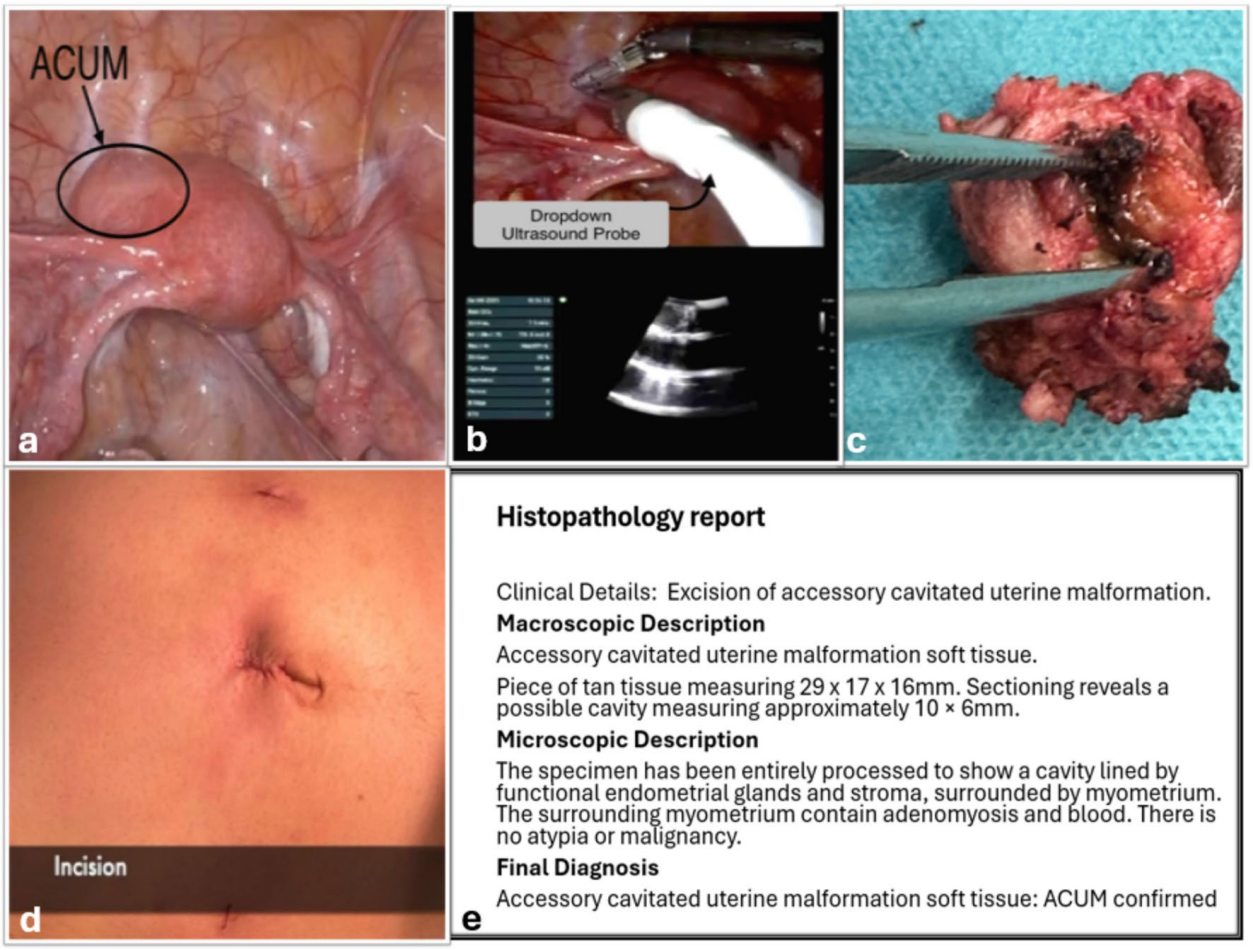
Intraoperative and pathological features of ACUM. **a** Intraoperative picture of ACUM in-vivo. **b** Robotic integrated intraoperative ultrasound probe. **c** Gross specimen showing endometrial cavity within the myometrium. **d** Port-site surgical incisions. **e** Histopathological confirmation of functional endometrial tissue within myometrium

Bipolar fenestrated forceps were introduced through one assistant port, while the robotic-integrated intraoperative ultrasound probe (BK Medical robotic transducer using TelePro vision) was introduced through the other assistant port and applied directly onto the uterine surface. Real-time sonographic mapping delineated the exact location, depth, and margins of the accessory cavitated lesion in relation to the endometrial cavity and serosal surface of the uterus (Fig. 2).

The robotic output was displayed on the vision cart, where the screen was divided into two sections: the upper half showing the intraperitoneal cavity and the lower half displaying the transmitted ultrasound image.

The uterine serosa overlying the lesion was incised in a curvilinear manner using the monopolar scissors. Diluted vasopressin was injected into the overlying myometrium to minimize bleeding and facilitate hydro-dissection of the surgical plane, followed by a combination of sharp and blunt dissection in a circumferential manner until the lesion was completely separated.

Care was taken not to breach the endometrial cavity. Once the lesion was isolated and enucleated, the myometrial cavity was reconstructed in layers using knot-less self-anchoring barbed sutures. The drop-down ultrasound device was then used again to assess the integrity of the endometrial cavity.

### Postoperative course and clinical outcome

Postoperatively, the patient was discharged within 24 h as recovery was unremarkable. To facilitate uterine healing postoperatively, she was prescribed progesterone-only pill for a period of three months. Following completion of hormonal therapy, menstruation resumed spontaneously with complete resolution of symptoms. At follow-up, she remained asymptomatic with no recurrence of pelvic pain or dysmenorrhea.

## Discussion

ACUM was first described by Cullen in 1908. In 2010 Acien et al. formally termed and established the diagnostic criteria for this entity [5]. Despite increasing recognition, ACUM remains unclassified within the rASRM and ESHRE/ESGE classification systems, hence the ongoing issues with underdiagnosis and under-reporting of the condition [6, 7].

### Clinical presentation and diagnosis

ACUM predominantly affects nulliparous women aged 21–29 years with onset of symptoms from or after menarche [2, 8]. The hallmark presentation includes severe dysmenorrhea beginning shortly after menarche. In women presenting with lateralized chronic cyclic pelvic pain, ACUM should be considered in the differential diagnosis [9]. Symptoms progressively worsen and prove refractory to nonsteroidal antiinflammatory drugs (NSAIDs) and hormonal therapies [3, 10, 11].

Transvaginal ultrasonography is the first-line imaging modality used to diagnose ACUM. The ultrasound findings are characterized by a well-circumscribed, intramyometrial lesion beneath the round ligament insertion with “ground-glass” echogenic content [12]. The differential diagnosis of ACUM includes uterine fibroids, adenomyoma, and noncommunicating rudimentary horns. The accuracy of diagnosis can be improved with three-dimensional ultrasound [13, 14]. MRI remains the second-line investigation for preoperative diagnosis, demonstrating the characteristic three-layer concentric pattern: T1/T2 hyperintense endometrial lining, hypointense junctional zone, and normal outer myometrium [2].

### ACUM and its effects on fertility

The impact of ACUM on fertility remains unclear. A systematic review by Strug et al., documented infertility in 4.3% of 70 ACUM cases, with the authors concluding that “the impact of ACUM on infertility has not been described”. In ACUM patients with infertility, concomitant endometriosis has been frequently noted, confounding the attribution of infertility to ACUM [15].

Theoretical mechanisms of fertility impairment, similar to those in adenomyosis and endometriosis, have been proposed as ACUM contains functional endometrial tissue within the myometrium undergoing cyclical changes with hemorrhage. In endometriosis, the pelvic environment is hostile for gametes due to immune dysregulation with inflammatory cytokines causing damage to the ovaries and pelvic tissue; disrupting uterine function resulting in implantation failure [16]. Proinflammatory cytokines, including TNF-α, IL-6, IL-1β, IL-8, and IL-17 found in follicular and peritoneal fluid, create a hostile microenvironment that adversely affects endometrial receptivity and induces sperm apoptosis [16, 17]. Similarly, there is immune dysfunction with increased macrophage infiltration, elevated inflammatory cytokines with altered T-helper/T-regulatory cell ratios in adenomyosis [18]. Animal models by Bourdon et al., confirm that the proinflammatory environment during the implantation period directly reduces embryo implantation sites and alters placental development [19].

Whether ACUM similarly affects the adjacent normal endometrium through paracrine inflammatory signaling remains unknown. Although there are a few case reports that demonstrate spontaneous conception after ACUM excision, there is no clear evidence that resolution of local inflammation and reduction in pain facilitating intercourse following excision of ACUM results in spontaneous conception [15]. Prospective controlled studies evaluating fertility outcomes in ACUM patients with and without endometriosis are essential to clarify the effect of pro-inflammatory state on endometrial receptivity and conception. Such studies would also clarify whether the observed effect on reproductive function and infertility is attributable to ACUM only or the coexisting pathology.

### Management evolution

Medical management with NSAIDs, oral contraceptives, and GnRH agonists provides temporary relief with symptoms recurring after treatment discontinuation [20]. Currently, laparoscopic excision is the gold standard for definitive management with complete resolution of symptoms and restoration of normal uterine function [2, 21]. In 2023, a review of 70 cases demonstrated complete symptom resolution in 90.7% of patients, with no major complications. Surgical excision also facilitated spontaneous conception in patients with concomitant infertility, suggesting reproductive benefits beyond symptom control [15].

### Novel application of robotic-integrated intraoperative ultrasound

This case demonstrates an innovative technique that uses real-time sonographic mapping by a robotic-integrated intraoperative ultrasound method that enhances precise excision of ACUM. Such techniques have occasionally been used in gynecologic oncology surgery but have not been previously described for Mullerian anomalies. The high-resolution 3D visualization and improved dexterity in robotic surgeries provide enhanced precision in complex cases requiring meticulous dissection [22].

The integration of real-time intraoperative ultrasound with 7–13 MHz transducers adds a critical dimension to surgical decision-making. Using flexible drop-in probes via 8 mm trocars and maneuvering through robotic graspers allows surgeons to access anatomical sites that are not accessible through conventional laparoscopic ultrasound. This technology has proven invaluable for real-time tissue assessment, sentinel lymph node identification, and determining resection margins in gynecology oncology [23].

The application of this technology in surgical excision of ACUM addresses specific technical challenges inherent to this condition. This is due to the proximity to critical neurovascular structures including the round ligament, uterine vessels, and its deep intra-myometrial location. ACUM is significantly noted in young nulliparous women, which makes preservation of healthy myometrium for future reproduction through precise and complete excision important.

Robotic-integrated ultrasound enables:

1. Real-time lesion localization: Direct visualization of the exact borders and depth of accessory cavity within the myometrium helps to overcome limitations of preoperative imaging that may not accurately translate to the surgical field.
2. Tissue plane identification: Dynamic assessment of the interface between the wall of accessory cavity and surrounding normal myometrium facilitates meticulous dissection minimizing loss of healthy myometrium and enables complete excision.
3. Vascular mapping: Real-time identification of blood vessels supplying the myometrium surrounding the accessory cavity enables strategic vessel ligation and improved hemostatic control.
4. Completeness verification: Immediate confirmation of complete lesion excision potentially reduces recurrence risk and leads to complete resolution of symptoms.
5. Myometrial integrity assessment: Postexcision evaluation of myometrial thickness and integrity of endometrial lining facilitates appropriate closure to improve uterine strength and future pregnancy outcomes.

Conventional laparoscopic approaches rely solely on visual cues and tactile feedback, whereas integration of real-time ultrasound in robotic platforms provides significant advantages. While preoperative MRI provides excellent anatomical detail, intraoperative ultrasound offers real-time dynamic guidance during dissection that adapts to surgical manipulation and tissue distortion. The enhanced dexterity in a robotic platform allows optimal probe positioning and angulation, facilitating precise and complete excision in unreachable locations.

The translation of this technology from oncologic to benign gynecologic applications exemplifies how surgical innovation in one domain can address unmet needs in another. Although ACUM is rare and there are no standardized surgical protocols, the adoption of advanced intraoperative imaging may significantly improve surgical and reproductive outcomes with less surgical trauma to healthy myometrium.

### Alternative minimally invasive approaches

Marivel et al. described ultrasound-guided ethanol sclerotherapy, a non-surgical option done under local anesthesia, offering myometrial preservation [24, 25]. This technique has been found to be beneficial in selected patients seeking to avoid myometrial scarring or those with significant surgical risk factors. However, long-term data on sclerotherapy efficacy is limited, and surgical excision is the preferred definitive treatment and results in complete resolution of symptoms.

## Conclusions and future directions

The 2025 Euro-Chinese consensus provides standardized diagnostic and management guidelines for ACUM. While surgical excision remains the definitive treatment, the integration of real-time intraoperative ultrasound guidance in robotic platforms represents a meaningful technical advancement. This case demonstrates the potential advantages and flexibility of adapting oncologic surgical techniques to complex benign gynecologic conditions requiring precise surgeries.

Referral to specialized centers with advanced minimally invasive surgery facilities, including robotic platforms and intraoperative imaging, ensures optimal surgical outcomes with complete excision and preservation of reproductive function. Future research should evaluate whether robotic-integrated ultrasound reduces operative time, complication rates, and recurrence when compared to conventional laparoscopic approaches. Long-term studies examining fertility outcomes and pregnancy complications following ultrasound-guided robotic excision will determine whether this enhanced precision translates to improved reproductive function.

## Data Availability

No datasets were generated or analyzed during the current study.
